# Why We Eat the Way We Do: A Call to Consider Food Culture in Public Health Initiatives

**DOI:** 10.3390/ijerph182211967

**Published:** 2021-11-15

**Authors:** Edwina Mingay, Melissa Hart, Serene Yoong, Alexis Hure

**Affiliations:** 1 School of Medicine and Public Health, University of Newcastle, Callaghan, Newcastle, NSW 2308, Australia; syoong@swin.edu.au (S.Y.); alexis.hure@newcastle.edu.au (A.H.); 2 Hunter Medical Research Institute, New Lambton Heights, Newcastle, NSW 2305, Australia; 3 School of Health Sciences, University of Newcastle, Callaghan, Newcastle, NSW 2308, Australia; mel.hart@health.nsw.gov.au; 4 Hunter New England Mental Health Service, Waratah, Newcastle, NSW 2298, Australia; 5 Hunter New England Population Health, Wallsend, Newcastle, NSW 2287, Australia; 6 School of Health Sciences, Swinburne University of Technology, Hawthorn, Melbourne, VIC 3122, Australia

**Keywords:** food culture, nutrition, public health, health promotion, physical health, mental health

## Abstract

The way we eat has changed dramatically in only a few decades. While definitions of food culture have previously existed, a clear description of modern food culture that can be used for health promotion is lacking. In this paper, we propose a concept of food culture for application within public health, what a positive food culture looks like compared to negative elements that have dominated in developed countries and the consequences for physical and mental health and wellbeing. We support calls to action from the international community to reconsider the way we eat. All segments of society have a role to play in building a positive food culture, and it is critical that macro (policy and systems) and meso (community) level environmental factors align and provide supportive environments that promote health-enhancing behaviours. Defining food culture is a necessary step towards articulating the complexities that influence food behaviours and impact health. The ultimate goal is collective action to enable population-wide and sustained improvements to the way we eat, and how we think and feel about food.

## 1. Introduction

The way we engage with and consume food has changed dramatically in only a few decades with changes to food systems and environments that have exacerbated poor eating patterns and food choices [1]. The negative impact of these changes on physical and mental health and wellbeing at a population level is a global priority [2]. Dietary risk factors are driving the global burden of disease, including mental health, which have escalated in both developed and developing nations [2,3,4,5,6]. In 2016, more than 2.2 billion people worldwide were overweight or obese [7], and it has been projected that without change to current policies, global levels could increase to 3.28 billion people by 2030 (from 1.33 billion in 2005) which represents one third of the projected global population and increased burden of disease [2]. Globally in 2017, 11 million deaths and 255 million disability adjusted life years were attributable to dietary risk factors, in particular diets high in sodium, and low in fruit, wholegrains, nuts and seeds, vegetables, and seafood omega-3 fatty acids [8]. These findings provide a stark reminder of the significant relationship between diet quality and non-communicable diseases (NCDs) which has been examined extensively and is well recognised [8,9,10]. For example, diet is a primary risk factor for type 2 diabetes, which ranked as the ninth leading cause of mortality worldwide in 2017 (from eighteenth in 1990) and affected 462 million people (6.28% of the population); a prevalence rate of 6059 per 100,000 that is projected to rise to 7079 per 100,000 by 2030 [11]. Importantly, diet is a preventable risk factor, highlighting the need to improve dietary practices, with contributions from all segments of society [8]. Peak authoritative bodies, including the World Health Organization, are calling for a shift in, or at least a share of, focus from treating disease to promoting health and more sustainable food systems that deliver healthy diets for all and promote lasting health-enhancing behaviours [2,10,12,13].

In response to this, we call for an approach that directs focus towards a positive food culture that extends beyond individual level factors to include the influence of social, economic, technological and political factors that have re-shaped our foodways and changed habitual behaviours and beliefs (cultural considerations) around food and eating [14]. To date, efforts to improve healthy eating have largely focused on strategies in isolation that target behaviour change at an individual level [15,16]. However, without strategies that incorporate and target environmental, behavioural and cultural determinants to influence habitual food behaviours, values and beliefs, it is unsurprising that most of these strategies on their own show limited effects for health gains and longer-term efficacy [17,18]. The promise of current nutrition interventions in achieving population wide health gains is clearly not being achieved. It is time to act with vision and leadership, challenging traditional ways of improving public health nutrition and investing in strategies that are likely to benefit many, over time, and for future generations. We join Hedegaard (2016) and support the need to define and understand the vast and complex components that influence eating patterns and subsequently shape food culture [14], and Block et al. (2011) who propose a shift in paradigm towards ‘food as wellbeing’ to capture social and cultural considerations for our understanding of the role of food in our lives [19]. Food culture has always existed but has not been consistently defined. In this manuscript, we seek to define food culture to fill this gap, and in doing so, highlight its significance, and call for its application within public health. We explore the detrimental changes to food culture among developed countries, and highlight opportunities and examples where understanding what a positive food culture looks like can help improve the design and longer-term efficacy of nutrition-related health promotion efforts to ultimately improve habitual food behaviours, values and beliefs going forward.

## 2. Food Culture Explained

Culture within social anthropology has been described by Wolcott (2008) as “the various ways different groups go about their lives and to the belief systems associated with that behaviour” [20]. Applying this concept, here within, we refer to *food culture* as what we do, think and feel around food as an individual or group, within the social and environmental constructs at that time. Our food culture is influenced by food-related drivers that extend beyond individual factors to include our surrounding environments, food socialisation and cultural practices (people, place, policy and time) that interact (directly and indirectly), are highly influential for food choice, and shape the way we eat. Importantly, it encompasses cultivated and shared knowledge and behaviours through inherited ideas, and learning and accumulated experience throughout our lives that mould our beliefs and values around, and relationship with, food and eating. Food culture drivers include:

- *Our social milieu*: close relationships and extended influencers from the media; our interactions, behaviours, ways of thinking and understanding of food, that create social norms through exposure and accumulated experience [21].
- *Place*: physical settings within the home, workplace, neighbourhood, educational settings that we occupy to engage with and consume food.
- *Guidelines*: rules, expectations and instructions within a society that guide people around food-related behaviour [21].
- *Food literacy*: cultivated and transmitted food literacy across generations, influenced by temporal (perception of time) and spatial (perception of physical space) dimensions, cultural practices, economic resources, and habitual behaviours linked to global and traditional changes to food procurement, selection, preparation and consumption [22,23].
- *Food systems*: the activities that encompass paddock to plate to disposal practices, shaped by policy, economics, and health, ethical and sustainability concerns [2,24].

Food culture expresses identity and meaning, links to dietary patterns, and therefore impacts health and wellbeing. It has always existed, and elements have been explored and described; particularly within sociology, public health literature and recent dietary guidelines [21,25,26,27]. Parallel influences can be drawn from the health promotion and public health literature, including policy and ecological frameworks [1,27,28,29,30], and principles from the Ottawa Charter [31] and the Constitution of the World Health Organization [32] that remain firmly relevant today.

Our food culture is closely linked to our surrounding food environments. We argue for the application of food culture within public health to expand the lens and consider cultural and symbolic meanings around food and eating within our food environments. Applying a food culture lens forces the opportunity to question how food culture is represented within each environment where we engage with and consume food. Is it one that considers and promotes a positive and supportive approach to food behaviours, and contributes to moulding positive values and beliefs around food and eating for individuals and communities? Or is it one that diminishes the vital contribution food plays in our lives that encompasses a broad umbrella over health, wellbeing, socialisation, knowledge and skills, access and availability, values and beliefs.

Exploring food culture in the way we approach nutrition interventions enables a holistic picture of the complexities that shape our food behaviours within society, the structure that provides organisation for people. This includes characteristics of all segments of society (individuals, families, communities, businesses, industries, organisations, governments) to build our understanding of why we eat the way we do. Food behaviours *“are not universal, natural or inevitable”* [21], nor are they static. This challenges us to think about the whole of food culture being greater than the sum of its parts.

## 3. Detrimental Changes to Food Culture

The way we engage with and consume food has changed. Globalisation of food, urbanisation, information technology, social and lifestyle changes all contribute to moulding the environment in which we live. It is suggested that these changes have played a significant role in shaping the population’s eating behaviours, and therefore the risk and burden of non-communicable conditions, including mental health [5,14,27,33].

The globalisation of food has impacts on food choices, habitual food behaviours and nutrient intake. Our modern food systems are characterised by inequitable availability and accessibility to safe and nutritionally adequate food [2]. More foods than before have been manufactured, refined, repackaged and branded. Choice has expanded with increased imports, abundant convenience and ultra-processed foods [5,34]. For the most part, this has led to poorer nutritional quality alternatives than the original wholefoods [5,35,36]. In supermarkets and convenience stores, low-nutritional quality food at low cost is readily available and heavily marketed, often targeting vulnerable groups [37,38]. Super-sized portions, portable foods and beverages, and take-away meals have displaced social and cultural functions of the home-prepared meals that were typically shared around the dining table [5].

Urbanisation and increased parental workforce hours have increased time away from home and changed the way lives are structured [39]. Time constraints faced as a result demand time-saving food sourcing and preparation towards convenience foods that are associated with poorer nutritional quality [36,40]. Population level evidence indicates urban populations consume more meals away from the home environment [39]. We hypothesise such social changes have decreased the transfer of food knowledge and skills from family and carers to younger generations, including a loss of skills, value, celebration and ritual around food.

The information age brings information overload and quickly spread exposure to socio-cultural influence (norms and values) and Western ideals. Competing nutrition messages and body misrepresentations through all forms of media is commonplace, creating confusion about food choices and body image, and increasing the risk of disordered eating patterns [41,42]. This includes idealised body shapes with unrealistic body fat composition or muscular physique. Body dissatisfaction and disordered eating behaviour are now common across social class, age and gender [43,44,45]. At the same time, there is increasing noise about ‘diets’, ‘obesity’ and the ‘thin ideal’. There has been an explosion of weight loss, or fad diets and products that are often commercially driven. They promise a quick fix without supporting evidence, and may compromise essential nutrient intake, organ function and ongoing health [46,47]. Without adequate media and food literacy, this poses challenges for younger generations to navigate, develop and practice health-promoting behaviours. The voice of reason, founded on scientific evidence, and positive values around food and eating gets lost amongst the noise of sensationalised media and marketing.

## 4. Health Promotion, Not Disease Deficit

The detrimental changes to food culture highlight a need for public health initiatives to focus on promoting healthy food-related behaviours, as a whole, to predominate, and change the way we think and feel about food and eating. The financial cost and intangibility of outcomes, has often led to a lack of investment in health promotion that facilitates a positive food culture, while significant investment in curative approaches continue [48]. Authorities are urging for greater investment, arguing initiatives that promote health-enhancing behaviours is offset by the reduced cost in treating disease [48].

Recurring themes resonate throughout calls to action from the international community to drive commitment towards food-related action to improve population-wide health. This includes calls for policy action, a multi-sectoral approach, creation of health-enhancing environments, regulatory action, investment, education and information, community awareness, early intervention, a life course approach and targeted efforts for priority populations [3,13,49,50]. In addition, climate change and the greenhouse gas contributions from farming practices and food production, demand a new approach to foodways and our attitudes around food and eating [12]. The United Nations (UN) Decade of Action on Nutrition 2016–2025 [13], and more recently, the EAT-Lancet Commission on Healthy Diets from Sustainable Sources [3] are important initiatives that align with the UN Sustainable Development Goals and call for healthier, more sustainable dietary intakes that are accessible for all.

Advances in dietary guidelines reflect the need for a fresh approach, highlighting the importance of environmental and policy interventions that promote and direct people and populations towards knowledge building, and practicing health-enhancing behaviours. The recently updated *Dietary Guidelines for Americans 2020–2025,* provides a public health framework that promotes continuity of healthy eating patterns (as a whole rather than isolating foods and nutrients) across different life stages, recognising the benefits of developing healthy habits for life course disease prevention. The guidelines focus on nutrient-dense options across food groups to meet nutritional needs, which can be customised to reflect personal preferences, cultural traditions, and budgets; the focus is on health promotion across multiple settings, not disease deficit [51]. The guidelines have been said to fall short in addressing the link between dietary practices and planetary health [52], though they do recognise the important contribution from all segments of society to support healthy choices.

Other examples include the *Dietary Guidelines for the Brazilian Population* which adapts a social-ecological model to illustrate the need for collective action; recognising everyone has a role to play to promote healthy eating practices. The principles focus on fresh or minimally processed foods, social and cultural dimensions of food choice, modes of eating (time, focus, place and company), environmental sustainability and the right to adequate and healthy food; the overall emphasis is the participation of all [26]. The *Canadian Dietary Guidelines 2019* extends healthy eating recommendations beyond the numbers on the plate to include consideration to food behaviours (where, when, why and how we eat). This includes mindful eating, cooking more often, enjoying your food, eating with others, and the benefits of learned and shared skills from others [25]. This is consistent with our description of food culture above.

## 5. Opportunities and Potential Solutions to the Current Challenges

A positive food culture aims to preserve and nurture good health and wellbeing, and promotes positive food behaviours, values and beliefs through both collective and independent efforts from each segment of society. Producing sustained change to the way we think and feel about food and eating is indeed challenging and ambitious. Multi-strategy opportunities and potential solutions are sought to achieve incremental gains across multiple levels, that are interconnected. To this effect, the World Cancer Research Fund developed the NOURISHING Framework which is an example of a viable tool to guide action across multiple levels to improve dietary behaviours and prevent obesity and NCDs. The framework identifies three domains (food environment, food system, behaviour change communication) and ten accompanying policy areas that can be adopted to suit populations’ varying community and national contexts [53]. In addition, it is a valuable resource that includes a database of initiatives that have been implemented around the world.

The aim of building a positive food culture is to consolidate the incremental gains, generate momentum and ultimately impact habitual change across communities, households and individuals alike. Opportunities and potential solutions include, but are not limited to:

- *Government and peak authoritative bodies*: policy, priorities and dietary guidelines to align around positive food culture, promoting a common goal [54]. A positive food culture could be placed at the forefront as a key construct in dietary guidelines and policy development; importantly, to foster public trust and provide supportive environments that promote health-enhancing behaviours and sustainable practices [5,52].
- *Educators*: to align teaching material with consistent, evidence-based food and nutrition recommendations in conjunction with environmental impacts and promotion of healthy body image. Schools provide an ideal platform to promote positive food behaviours among young people, and build knowledge, skills, confidence and media literacy [55,56,57]. For example, Australian initiatives targeting schools include the *Stephanie Alexander Kitchen Garden Program* which delivers interactive and hands-on food education with the aim to build positive and pleasurable food habits for life [58,59], and the recently launched *Butterfly Body Bright* promoting positive attitudes and behaviours around eating and our bodies [60]. Both initiatives endeavour to influence values and beliefs around food and eating.
- *Physical settings that provide a food service*: settings such as schools, childcare, workplace, recreational facilities, community programs, retail, restaurants, and catering to offer appealing dining spaces or environments that encourage positive food-related behaviours. For example, table displays, presentation and layout of food, and health-promoting menus and messaging. These are examples of behavioural economics principles that have been implemented in a range of dining settings that ‘nudge’ people towards healthier food selection and consumption [61,62,63,64,65,66]. In particular, health promoting schools have the opportunity to reach large numbers of students, provide health-enhancing environments, and reduce disparities [67]. For example, adopting behavioural economics principles in the United States, the *Smarter Lunchroom Movement* offers schools a suite of low or no-cost evidence-based strategies to promote healthy school lunch options and reduce food waste [68,69]. School meal programs around the world contribute to social cohesion, and aim to improve school attendance and provide access to nutritionally balanced meals [70].
- *Food systems*: to prioritise the accessibility and affordability of safe and nutritionally adequate food for all people, with consideration to environmental impacts, cultural and traditional practices, and prioritising wholefoods over processed foods [2,3,5,36]. We can turn to the multi-layered nature of the Mediterranean Diet and the extensive literature that has exposed health benefits, enhanced quality of life, low environmental impacts and positive food values and behaviours [71,72]. The development of the Med Diet 4.0 framework and an updated Mediterranean Diet Pyramid have ensued, incorporating sustainability and environmental food system considerations alongside nutrition and health needs of populations and individuals [71,73].
- *Food literacy*: programs across a range of settings (for example, local communities, families and schools) to be promoted and evaluated with the goal to improve food and nutrition knowledge, hands-on skills, confidence and decision making around food selection and preparation. Longitudinal studies of cooking skills have indicated sustained skills, and positive outcomes around confidence and eating behaviours [74,75,76,77].
- *Marketing and media*: to prioritise the promotion of healthy body image, food choices and eating behaviours using appropriate language and messaging; an important medium for promoting positive attitudes around healthy eating and body image. Exposure to ideal body images, prescriptive dieting, and manipulative food marketing for general populations should be minimised. For example, an intervention designed to target adolescent values (autonomy from adult control and desire for social justice) and reframe food marketing to reject junk food in favour of healthy alternatives, found sustained change in dietary attitudes and food choices [37]. Without socio-cultural changes to what is portrayed in the media, realistic and positive body image representations and longer-term healthy eating behaviours will be difficult to achieve.
- *Home environment*: positive food behaviours in the home to be demonstrated and encouraged. The home environment plays a significant role in developing food literacy and habitual behaviours [78]. Importantly, behaviours can track from childhood and adolescence to adulthood [79,80,81], and across generations [82]. The need for a healthy start to life and the first 1000 days is well recognised [83]; and the subsequent 7000 days should not be underserved, but rather early gains secured with continued focus on building healthy behaviours during the transition to adulthood [84]. Raising children and adolescents within a positive food culture is one component of this.
- *Organisations and community groups*: continued efforts from groups to combat the degradation of wholefoods. For example, the Slow Food Movement, local farmers markets, community gardens and food festivals [85,86,87]. While these initiatives are considered niche rather than mainstream, they promote a hands-on approach where wholefoods and socialisation around food is celebrated. They build knowledge, skills and confidence, and empower people and communities to connect with food and expand their exposure and experience. However, it is recognised there are economic and physical determinants that influence affordability, availability, and accessibility to such opportunities. Barriers may include low income and food literacy, availability of food assistance programs in different countries, geographical locations where people live and associated neighbourhood food environments, transport links, environmental conditions, and seasonality of food. An example of efforts to overcome access and improve food culture for those on low income, in the United States it is recommended that farmers markets are expanded to multiple settings, and food assistance programs such as SNAP (Supplemental Nutrition Assistance Program) and WIC (Special Supplemental Nutrition Program for Women, Infants and Children) extend benefits to farmers markets purchases and offer related nutrition education [88].

Cultural considerations in the public health nutrition sphere requires changing beliefs, values and attitudes towards healthy eating patterns, which are vital for the longevity, and transfer of shared and learned food behaviours across generations; “to exist with some permanency through time and across space” [89]. The suggestions above aim to influence our belief systems and behaviour patterns towards sustained change and a positive food culture. There remains heavy work towards disseminating the importance and practice of sustainable diets alongside healthy food choices and behaviours, which reinforces the significance of food culture within population and planetary health for further consideration.

## 6. Conclusions

Understanding our current food culture is necessary to articulate the complexities that influence our food behaviours, values and beliefs, and have important implications for physical and mental health and wellbeing. Food culture provides the rationale to target multi-strategy multi-level nutrition interventions that incorporate environmental, behavioural and cultural elements to influence habitual food behaviours, values and beliefs. What is clear is that at a population level we need to foster health-promoting and supportive environments to enable population-wide improvements to the way we eat, and how we think and feel about food and our bodies.
